# Dopamine D_2_-Like Receptors Modulate Unconditioned Fear: Role of the Inferior Colliculus

**DOI:** 10.1371/journal.pone.0104228

**Published:** 2014-08-18

**Authors:** Amanda Ribeiro de Oliveira, Ana Caroline Colombo, Sangu Muthuraju, Rafael Carvalho Almada, Marcus Lira Brandão

**Affiliations:** 1 Laboratório de Neuropsicofarmacologia, FFCLRP, Universidade de São Paulo (USP), Ribeirão Preto, São Paulo, Brazil; 2 Instituto de Neurociências e Comportamento, INeC, Ribeirão Preto, São Paulo, Brazil; Medical University of South Carolina, United States of America

## Abstract

**Background:**

A reduction of dopamine release or D_2_ receptor blockade in the terminal fields of the mesolimbic system clearly reduces conditioned fear. Injections of haloperidol, a preferential D_2_ receptor antagonist, into the inferior colliculus (IC) enhance the processing of unconditioned aversive information. However, a clear characterization of the interplay of D_2_ receptors in the mediation of unconditioned and conditioned fear is still lacking.

**Methods:**

The present study investigated the effects of intra-IC injections of the D_2_ receptor-selective antagonist sulpiride on behavior in the elevated plus maze (EPM), auditory-evoked potentials (AEPs) to loud sounds recorded from the IC, fear-potentiated startle (FPS), and conditioned freezing.

**Results:**

Intra-IC injections of sulpiride caused clear proaversive effects in the EPM and enhanced AEPs induced by loud auditory stimuli. Intra-IC sulpiride administration did not affect FPS or conditioned freezing.

**Conclusions:**

Dopamine D_2_-like receptors of the inferior colliculus play a role in the modulation of unconditioned aversive information but not in the fear-potentiated startle response.

## Introduction

Several studies have suggested modulatory roles for γ-aminobutyric acid (GABA), serotonin, opioid peptides, excitatory amino acids, and neuropeptides in the so-called encephalic aversion system (EAS), which includes the dorsal periaqueductal gray (dPAG), deep layers of the superior colliculus, amygdala, medial hypothalamus, and inferior colliculus (IC) [1]–[4]. However, little is known about the role of dopamine (DA) in the mediation of aversive states in the EAS.

The IC is a primary acoustic structure of the brainstem and the most caudal structure of the EAS that integrates sensory information of an aversive nature. Chemical and electrical stimulation of the IC causes unconditioned defensive behavior [2], [3], [5], [6]. We showed that fear-evoking stimuli increase the magnitude of auditory-evoked potentials (AEPs) that were directly recorded from the ventral aspects of the IC in response to an aversive auditory stimulus (AAS) [7], [8]. Local application of the excitatory amino acids glutamate and *N*-methyl-d-aspartate in the central and external nuclei of the IC also increased the acoustically evoked and spontaneous firing of most neurons in this region [9], [10].

Studies of DA-mediated mechanisms of fear/anxiety have considerable relevance with regard to the organism's reactivity following exposure to external acute stressors [11]–[14]. However, investigations of DA's mediation of the defense reaction in the midbrain tectum have been scarce. Aversive stimulation of the midbrain tectum at the escape threshold enhances DA release in the prefrontal cortex [15], and intracollicular injections of haloperidol, a nonselective DA receptor antagonist, enhance AEPs recorded directly from the IC [16]. These results support a previous study that showed that systemic injections of the DA D_2_ receptor antagonist sulpiride enhanced escape responses in rats subjected to a switch-off procedure [17]. In contrast, the association between changes in DA transmission and conditioned fear has been previously demonstrated in numerous studies. Thus, recent studies demonstrated that systemic and intra-amygdala injections of sulpiride attenuated the expression of conditioned fear [18]–[20].

As indicated above, numerous studies have reported that the activation of DA pathways that arise from the ventral tegmental area (VTA) and project to structures of the mesolimbic system increases learned anxiety, but the role played by DA mechanisms in innate fear is not entirely understood. Based on previous evidence from this laboratory, DA that acts through D2 receptors may play a distinct role in mediating the processing of aversive information of an unconditioned nature. To test this hypothesis, we microinjected sulpiride into the IC and evaluated exploratory behavior in rats in the elevated plus maze (EPM), which assesses the fear of animals to height and open spaces. We also recorded AEPs in response to aversive stimulation (loud sounds) recorded directly from electrodes implanted in the ventral aspects of the IC, an area of the IC that contains the neural substrates of fear [21] and as such would be associated with the processing of aversive information [8]. Furthermore, we evaluated conditioned fear by assessing fear-potentiated startle (FPS) and freezing responses to a light (conditioned stimulus) that was previously paired with footshock during training sessions. When the rats were returned to the test chamber and presented with startle-eliciting stimuli in either the presence or absence of the light, startle amplitude in light-startle trials (fear-potentiated startle) was significantly greater than startle amplitude in startle-alone trials (baseline).

## Methods and Materials

### Animals

A total of 206 male Wistar rats, weighing 250–300 g, from the animal facility of the University of São Paulo at Ribeirão Preto were used. The animals were housed in groups of four in plastic boxes (40×33×26 cm) and maintained under a 12 h/12 h light/dark cycle (lights on at 7:00 AM) at 23±1°C. The rats were allowed free access to food and water throughout the experiment. The experiments were performed during the light phase of the cycle. All of the experiments received formal approval (Process n°. 10.1.595.53.7 and 12.1.909.53.3) from the Committee on Animal Research and Ethics of the University of São Paulo.

### Surgery

The animals were anesthetized with a mixture of ketamine/xylazine (100/7.5 mg/kg, intraperitoneal) and fixed in a stereotaxic frame (David Kopf Instruments, Tujunga, CA, USA). The upper incisor bar was set 3.0 mm below the interaural line, such that the skull was horizontal between bregma and lambda. Guide cannulae (0.6 mm outer diameter) for drug injections and/or a brain electrode (160 µm diameter) were implanted into the midbrain, aimed at the IC. The guide cannulae and electrodes were introduced vertically using the following coordinates, with lambda as the reference: anterior/posterior, −1.0 mm; medial/lateral, ±1.5 mm; dorsal/ventral, −4.0 mm [22]. The cannulae and electrodes were fixed to the skull with acrylic resin and two stainless-steel screws. Each guide cannula was sealed with a stainless steel wire to protect it from blockage. Afterward, the rats were allowed 5 days to recover from the surgical procedure.

### Elevated plus maze

An EPM, described in detail elsewhere [23], was used, consisting of two open arms (50×10 cm) crossed at right angles with two closed arms of the same size. The two closed arms were enclosed by 40 cm high walls, with the exception of the central part of the maze (10×10 cm) where the open and closed arms crossed. The entire apparatus was elevated 50 cm above the floor. The experimental room was illuminated (30 lux). The experimental sessions were recorded by a video camera interfaced with a monitor and a DVD recorder in an adjacent room. Five days after surgery, the rats received intra-IC bilateral administration of dopaminergic drugs or vehicle. After 15 min, they were individually placed in the central area of the EPM with their nose facing one of the closed arms. The rats were then allowed to freely explore the maze for 5 min. Conventional EPM measures were recorded, including the number of entries into the closed and open arms and time spent in the open arms [24]. Additional behavioral categories were evaluated as previously described, including grooming, scanning, head dipping, rearing, end-arm exploration, peeping out, flat-back approach, and stretched-attend posture [24], [25].

### Auditory-evoked potentials

An experimental wire mesh cage (19×13.5×9 cm), located inside an insulated Faraday system and surrounded by a ventilated sound-attenuating plywood chamber (64×60×40 cm), was used. A loudspeaker located 10 cm behind the cage delivered continuous background noise (55 dB). Acoustic stimuli (100 clicks, 50 ms duration; 3000 Hz square-wave pulses) presented at a rate of 0.33 Hz (one every 3 s) were delivered via two piezoelectric speakers (12 Ω, 200 W; LeSon, São Paulo, SP, Brazil) mounted on the lateral walls of the sound-insulating box, 15 cm from the wire mesh cage. The acoustic stimulus was a pure tone (92.5 dB sound pressure level). Software and an appropriate interface (Lynx Electronics, São Paulo, SP, Brazil) controlled the presentation and sequence of the acoustic stimuli. Sound pressure levels were measured at the level of the ears of the animals using a 0.125 inch microphone and type 2636 DK-2580 measuring amplifier (Bruel and Kjaer Sound & Vibration Measurement A/S, Naerum, Denmark).

Brainstem AEPs are very small electrical voltage potentials that are recorded from electrodes in response to a repetitive stimulus along a specific brainstem auditory pathway. These potentials reflect neuronal activity in the auditory complex, mainly in the cochlear nucleus, superior olive, and IC [26]. Previous studies have shown that AEPs generated in the IC are sensitive to aversive manipulations [7], [27], [28]. The average value was obtained at the end of the sessions. Auditory-evoked potentials were recorded as the voltage difference between the tips of an insulated wire (150 µm) inserted through the cannula and tip of the guide cannula implanted in the IC. This voltage difference was fed into a TX001 amplifier (20–200 Hz bandwidth; Lynx Electronics, São Paulo, SP, Brazil) through two noiseless shielded cables that were passed through a hole in the roof of the Faraday cage. A previous study from our laboratory indicated no hemispheric differences in AEPs recorded under the present experimental conditions [27]. The output of the amplifier was connected to one of four channels on an analog/digital converter (CAD 12/36). The filtering, amplification, and digitalization of the signals were performed using the Sysdin system (Lynx Electronics, São Paulo, SP, Brazil). The potential signals were filtered (high-pass filter, 20 Hz; low-pass filter, 200 Hz) and sampled at a rate of 0.33 kHz. Sysdin software was programmed to sum individual AEP amplitudes. The data acquisition sweep began 10 ms before the onset of the sound stimulus (i.e., the latency to switch on the sound plus sound propagation) and continued for 200 ms after termination of the sound stimulus. During recording, the animals were monitored via a camera system placed in the experimental room. N1 was visually identified as the first negative wave, and P1 was identified at the first positive wave approximately 15 ms after the sound presentation. The positive peak P1 is considered an early component of the collicular response. Its amplitude was measured peak to peak, with peak latency between 5 and 8 ms [29]. The AEPs elicited from the IC were recorded from the ventrocaudal portions of the nucleus. This method of analysis is similar to previous studies from our and other laboratories [16], [29]–[31]. Peak amplitudes were defined as the maximum amplitude measured between N1 and the end of P1, similar to previous studies from our laboratory [7], [27], [31]. The computer output was graphically displayed on an XY plotter (Hewlett-Packard, Palo Alto, CA, USA).

### Fear-potentiated startle

#### Matching

The test cages were two wire-grid cages (16.5×7.5×7.5 cm) fixed to response platforms by four thumb screws each. The cages and response platforms were located inside ventilated, sound-attenuating plywood chambers (64×60×40 cm). A loudspeaker located 10 cm behind the test cages delivered both the startle stimulus (100 dB, 50 ms burst of white noise) and continuous background noise (55 dB). The startle reaction of the rats generated pressure on the platform, and the analog signals were amplified, digitized, and analyzed by Startle Reflex software (Med Associates, St. Albans, VT, USA). The startle reaction was recorded within a time window of 100 ms after the onset of the startle stimulus. For the first 2 days, the rats were placed in the test cage for a 5 min habituation period and afterward received a total of 30 startle stimuli with an interstimulus interval of 30 s. Each matching session was 20 min in duration. The rats were assigned to the different control and drug groups, such that each group had similar average startle amplitudes based on the last matching day.

#### Training

After recovery from surgery, the rats were conditioned to a light conditioned stimulus (CS) in cages (20×20×25 cm) with stainless-steel sides and back walls, transparent Plexiglas ceilings and front doors, and grid floors that consisted of stainless-steel rods. These cages were located within ventilated and sound-attenuated chambers (45×45×45 cm) that were different from the test cages to avoid conditioning to the context. The rats were placed in the training cage and received 10 CS-unconditioned stimulus (US) pairings after an habituation phase of 5 min using a 4 s, 6 W light CS that co-terminated with a 1 s, 0.6 mA footshock US. The intertrial interval varied randomly between 60 and 180 s. The duration of each training session was approximately 25 min.

#### Testing

The test session was conducted without footshock presentation in the same cages that were used for matching. Twenty-four hours after training, the rats received an intra-IC injection of sulpiride or vehicle and were placed in the startle test cage 5 min later. After 5 min of habituation, the rats received 60 startle stimuli (noise bursts) with a 30 s interstimulus interval. Half of the startle stimuli were presented in the absence of the CS (i.e., noise-alone trials) to provide a baseline, and the other half of the startle stimuli were presented in the presence of the CS (i.e., light-noise trials). In the light-noise trials, the startle stimulus was presented during the last second of a 4 s light presentation. The noise-alone and light-noise trials were interspersed randomly. The duration of the test session was 37 min.

### Drugs

The drugs, doses, and injection times were based on previous studies from our laboratory [19], [32]. The drugs used were (−)-quinpirole hydrochloride (D_2_ agonist), (*S*)-(−)-sulpiride (D_2_ antagonist), (±)-SKF-38393 hydrochloride (D_1_ agonist), and *R*-(+)-SCH-23390 hydrochloride (D_1_ antagonist). All of the drugs were purchased from Sigma (St. Louis, MO, USA) and dissolved in physiological saline (0.9%) shortly before use, with the exception of sulpiride that was first mixed with 2% Tween 80 (Sigma, St. Louis, MO, USA). Physiological saline or saline plus 2% Tween were used as vehicle control. Doses of 0.5, 1.0, 2.0, 3.0, and/or 4.0 µg were microinjected in a constant volume of 0.2 µl/site into the IC.

### Microinjection procedure

The injection needles were thin dental needles (0.3 mm outer diameter) connected to 5 µl Hamilton syringes with polyethylene tubes (Becton-Dickinson, Franklin Lakes, NJ, USA). Each rat remained free in a polypropylene box (28×17×13 cm), and the needles were introduced into the guide cannula until its lower end reached 1 mm below it. The solutions were injected into the IC in a volume of 0.2 µl using an infusion pump (Harvard Apparatus, Holliston, MA, USA). As noted previously, the radius of diffusion of a 0.5 µl volume is approximately 1.0 mm [33]. In the present study, we used a 0.2 µl drug volume, so the drug diffusion is presumed to be restricted to the target structure. Previous studies from our laboratory also showed that a volume of 0.2 µl did not spread in a tissue diameter greater than 0.4 mm [21], [34], so the drug injections in the present study unlikely spread beyond the target region. The microinjection procedure used here has been described in previous papers from this laboratory [18], [19], [20], [32].

### Histology

Upon completion of the experiments, the rats were anesthetized with a lethal dose of urethane (3 g/kg) and transcardially perfused with 0.9% saline followed by 4% formaldehyde. The brains were removed from the skulls and maintained in formaldehyde solution for 2 h, after which the brains were cryoprotected in 30% sucrose for 48 h. Serial 60 µm coronal brain sections were cut using a cryostat, mounted on gelatin-coated slides, and stained with Cresyl violet (5%; Sigma-Aldrich, St. Louis, MO, USA) to localize the positions of the microinjection sites according to the atlas of Paxinos and Watson [22].

### Statistical analysis

The data are expressed as mean + SEM. The conditioned freezing response was analyzed using Student's *t*-test. The EPM and AEP data were subjected to one-way analysis of variance (ANOVA). Two-way repeated-measures ANOVA was conducted on the FPS data. *Post hoc* differences were tested using the Newman-Keuls test. Values of *p*<0.05 were considered significant.

## Results

**Figure 1:**
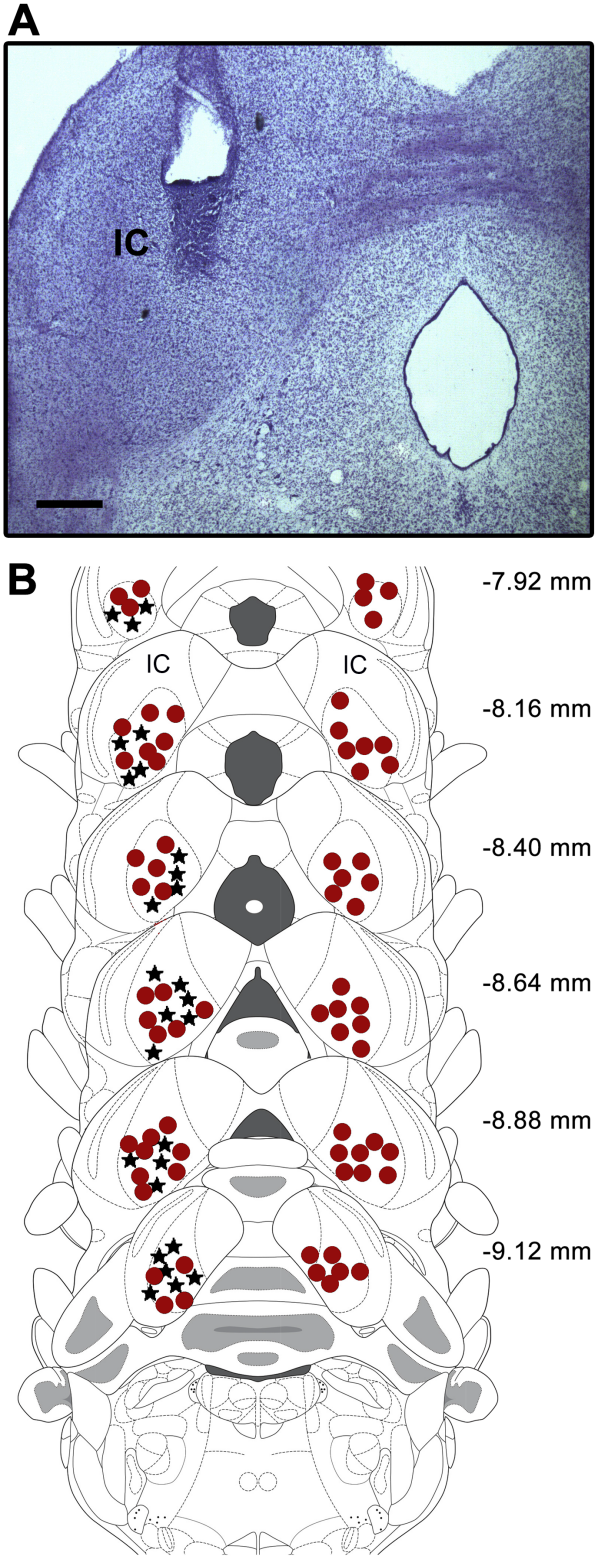
Target microinjection/recording sites in the inferior colliculus. (A) Photomicrograph showing a representative microinjection site in the IC. (B) Outlines of the microinjection and recording sites on coronal brain sections from the Paxinos and Watson atlas (2007) are shown. Symbols of each group were represented in the same side for the sake of clarity. • – injection sites, ★ – recording electrode implants. The number of points in the figure is less than the total number of rats used because several points overlap. Scale bar = 0.5 mm. IC = inferior colliculus.

All of the microinjections and recording sites in the present study were situated in the ventral aspects of the IC. Fig. 1 depicts the histological localization of the microinjection/recording sites in the IC, based on the diagrams in the atlas of Paxinos and Watson [22].

### Elevated plus maze

**Figure 2:**
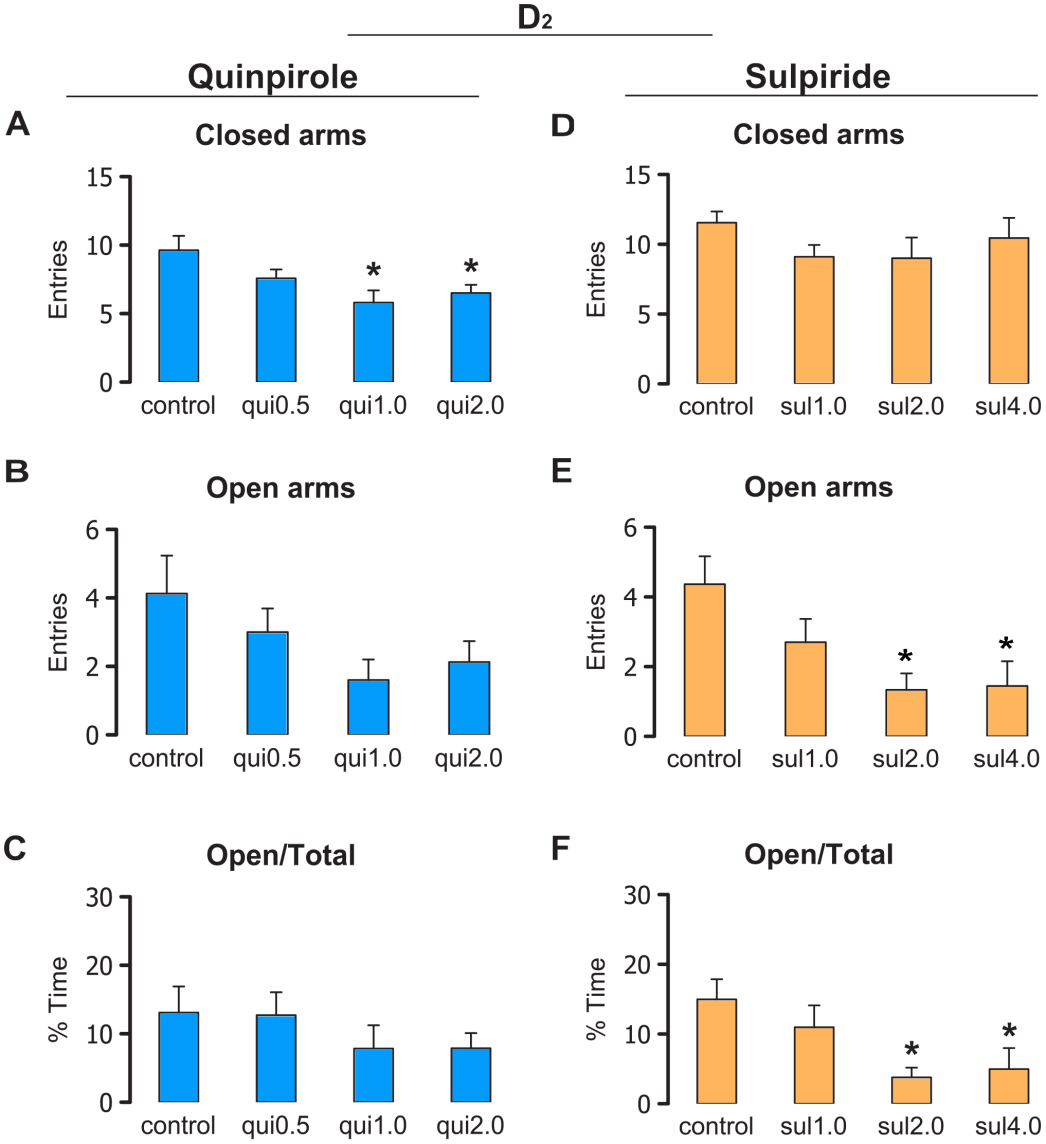
Effects of D_2_ drugs in the elevated plus maze. The figure shows the effects of intra-IC injections of the vehicle for quinpirole (Control; *n* = 8), 0.5 µg/0.2 µl quinpirole (*n* = 7), 1.0 µg/0.2 µl quinpirole (*n* = 10), 2.0 µg/0.2 µl quinpirole (*n* = 8), the vehicle for sulpiride (Control; *n* = 11), 1.0 µg/0.2 µl sulpiride (*n* = 10), 2.0 µg/0.2 µl sulpiride (*n* = 9), and 4.0 µg/0.2 µl sulpiride (*n* = 9) on exploratory behavior in rats subjected to the elevated plus maze. (A, D) Number of entries into the closed arms of the elevated plus maze. (B, E) Number of entries into the open arms of the elevated plus maze. (C, F) Percentage of time spent in the open arms relative to total time. The data are expressed as mean + SEM. *p<0.05, compared with control group (Newman-Keuls test).

Fig. 2A–C show the mean behavioral responses for rats that received intra-IC injections of vehicle (Control; *n* = 8), 0.5 µg/0.2 µl quinpirole (*n* = 7), 1.0 µg/0.2 µl quinpirole (*n* = 10), and 2.0 µg/0.2 µl quinpirole (*n* = 8) before the testing session in the EPM. The ANOVA did not indicate a statistically significant effect of treatment on the frequency of open arm entries (*F*_3,29_ = 2.14, *p*>0.05) or percentage of time spent in the open arms (*F*_3,29_ = 0.78, *p*>0.05). The ANOVA indicated statistically significant effects of treatment on the frequency of closed arm entries (*F*_3,29_ = 4.03, *p*<0.05). The Newman-Keuls *post hoc* test revealed a reduction of closed arm entries in the groups treated with 1.0 and 2.0 µg/0.2 µl quinpirole compared with the control group (*p*<0.05). The analysis of the complementary ethological categories also indicated a motor effect of quinpirole in the IC (Figure S1 in File S1).

Fig. 2D–F show the mean behavioral responses for rats that received intra-IC injections of vehicle (Control; *n* = 11), 1.0 µg/0.2 µl sulpiride (*n* = 10), 2.0 µg/0.2 µl sulpiride (*n* = 9), and 4.0 µg/0.2 µl sulpiride (*n* = 9) before being tested in the EPM. The ANOVA did not indicate statistically significant effects of treatment on the frequency of closed arm entries (*F*_3,35_ = 1.18, *p*>0.05) but revealed statistically significant effects of treatment on the frequency of open arm entries (*F*_3,35_ = 4.35, *p*<0.05) and percentage of time spent in the open arms (*F*_3,35_ = 3.69, *p*<0.05). The Newman-Keuls *post hoc* test revealed a reduction of open arm exploration in the groups treated with 2.0 and 4.0 µg/0.2 µl sulpiride compared with controls (*p*<0.05). The analysis of the complementary ethological categories in the EPM also indicated an anxiogenic-like effect of sulpiride in the IC (Figure S1 in File S1).

The mean behavioral responses for rats that received intra-IC injections of the vehicle for SKF-38393 (Control; *n* = 10), 1.0 µg/0.2 µl SKF-38393 (*n* = 9), 2.0 µg/0.2 µl SKF-38393 (*n* = 8), the vehicle for SCH-23390 (Control; *n* = 17), 1.0 µg/0.2 µl SCH-23390 (*n* = 10), 2.0 µg/0.2 µl SCH-23390 (*n* = 12), and 4.0 µg/0.2 µl SCH-23390 (*n* = 10) before being tested in the EPM did not show significant differences (Figures S2 and S3 in File S1).

### Auditory-evoked potentials

**Figure 3:**
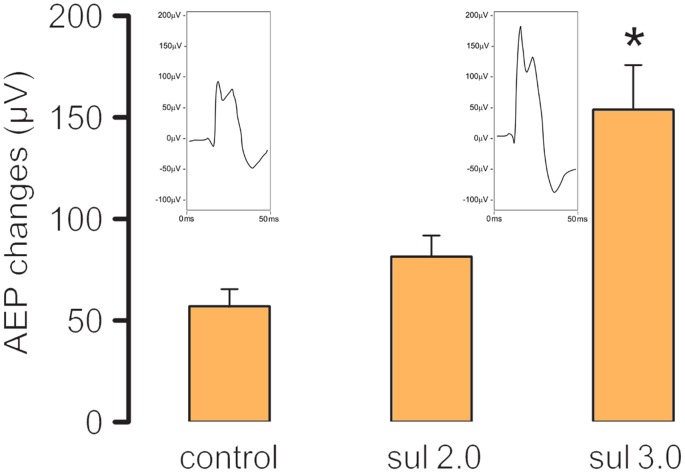
Effect of sulpiride on the auditory-evoked potentials. The figure shows the effects of intra-IC injections of vehicle (Control; *n* = 6), 2.0 µg/0.2 µl sulpiride (*n* = 7), and 3.0 µg/0.2 µl sulpiride (*n* = 8) on the amplitude of AEPs recorded in the IC. The data are expressed as mean + SEM. *p<0.05, compared with control group (Newman-Keuls test). Representative collicular AEPs recorded from the central and external nuclei of the inferior colliculus following local injections of saline (control) or 3.0 µg/0.2 µl sulpiride are also illustrated.

Fig. 3 shows the mean AEP changes recorded for rats that received an intra-IC injection of vehicle (Control; *n* = 6), 2.0 µg/0.2 µl sulpiride (*n* = 7), and 3.0 µg/0.2 µl sulpiride (*n* = 8) before AAS presentation. The one-way ANOVA revealed a significant effect of treatment (*F*_2, 18_ = 5.91, *p*<0.05). The Newman-Keuls *post hoc* test revealed that 3.0 µg/0.2 µl sulpiride significantly increased AEPs compared with the control group (*p*<0.05). In the present study, we also found that loud tones (92.5 dB, AAS), in contrast to tones presented at low intensity, increased Fos distribution in structures that are responsible for the integration of defense reactions to unconditioned aversive stimulation, such as the periaqueductal gray (Figures S4, S5, S6 in File S1).

### Fear-potentiated startle

**Figure 4:**
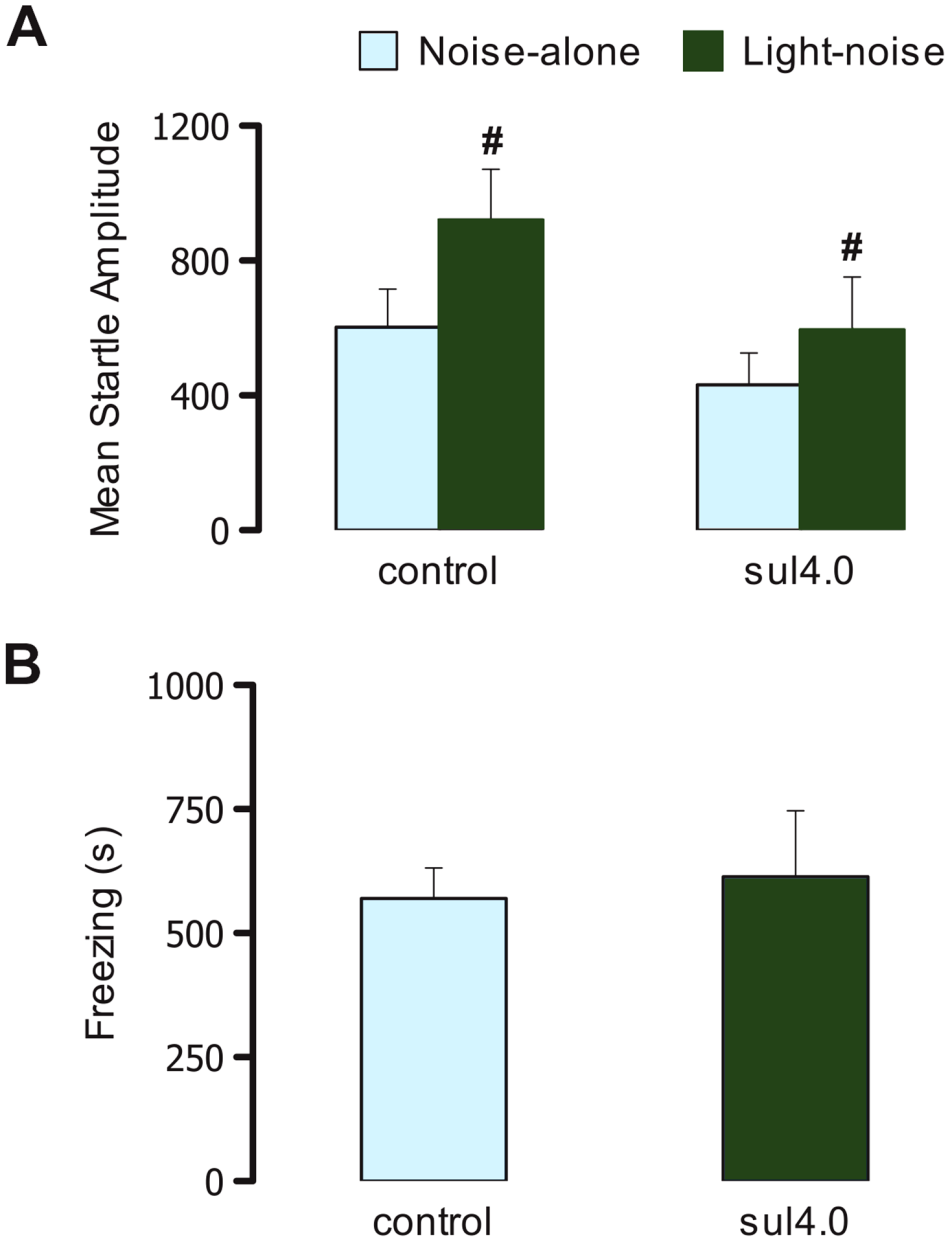
Effects of D_2_ drugs on fear-potentiated startle and conditioned freezing. The figure shows the effects of intra-IC injections of vehicle (Control; *n* = 12) and 4.0 µg/0.2 µl sulpiride (*n* = 11) on the mean startle amplitude (A) and conditioned freezing (B) in rats subjected to the conditioned fear test. The data are expressed as mean + SEM. ^#^ *p*<0.05, compared with noise-alone (Newman-Keuls test).

Fig. 4A shows the startle amplitude in rats that received intra-IC injections of vehicle (Control; *n* = 12) and 4.0 µg/0.2 µl sulpiride (*n* = 11). The two-way repeated-measures ANOVA revealed a significant effect of trial type (*F*_1,45_ = 14.27, *p*<0.05) but no significant effect of treatment (*F*_1,21_ = 2.02, *p*>0.05) and no treatment×trial type interaction (*F*_1,45_ = 1.51, *p*>0.05). The Newman-Keuls *post hoc* test revealed an overall enhancement of the startle response in light-noise trials compared with noise-alone trials (*p*<0.05). Fig. 4B shows the conditioned freezing response during the test session in the same rats. Student's *t*-test revealed no significant effects of intra-IC injections of sulpiride on the conditioned freezing response (*t* = 0.31, *p*>0.05).

## Discussion

GABA and excitatory amino acids have been shown to have opposing actions in the mediation of defensive behavior generated and organized in the IC [35]–[39]. Serotonin and opioid peptides play inhibitory roles in the neural substrates of aversion in this structure [3], [4], [36], [38]. Little is known about the role of DA in mediating unconditioned and conditioned fear in the IC. In the present study, local injections of the D2 receptor antagonist sulpiride into the IC in rats caused proaversive effects in the EPM and enhanced AEPs in response to loud sounds. We also found that local infusion of sulpiride into the IC did not affect the startle response (whether unconditioned or conditioned).

Although the precise neural circuitry of DA transmission involved in aversive states remains unclear, pharmacological and neurochemical studies appear to implicate prefrontal cortex [40], [41] and nucleus accumbens DA terminals in the response to acute stressors [42]–[49]. With regard to DA's mediation of conditioned fear, an increase in DA metabolism in the mesolimbic system is correlated with conditioned fear, and a decrease in DA activity in the basolateral amygdala causes a reduction of the expression of conditioned fear responses [19], [20], [34], [50], [51]. In fact, intraperitoneal injections of low doses of the D_2_ receptor agonist quinpirole act at autoreceptors on VTA neurons, slowing DA release at their terminals and also causing a reduction of conditioned fear responses [19], [34], [51]. Therefore, the fear response to a light-CS appears to depend on the activation of mesolimbic dopaminergic connections and can be specifically modulated by manipulating these projection neurons. In the present study, intra-IC sulpiride injections did not affect FPS or conditioned freezing in response to a light-CS.

With regard to filtering processes associated with aversive information, accumulating evidence suggests the involvement of DA mechanisms in structures of the EAS. Although DA-mediated mechanisms that are involved in the generation of aversion at the level of the IC have not been extensively studied, this region has appreciable dopaminergic activity [52], [53]. In the present study, intra-IC injections of sulpiride exerted clear anxiogenic-like effects in rats subjected to the EPM and enhanced the amplitude of AEPs in response to loud auditory stimuli recorded from the ventral aspects of the IC. The lack of effects of intra-IC injections of the D1 receptor agonist SKF-38393 and antagonist SCH-23390 in rats in the EPM is consistent with the much higher density of D2 receptors than D1 receptors in the IC [53].

A question that arises is the way in which the IC is involved in DA-mediated fear. The IC is distinguished from other auditory centers in the brainstem by its connections with motor systems [54]. Support for a functional link between the activation of these neurons and behavioral responses induced by aversive stimulation of the IC has also been reported [15]. According to several studies, the IC is positioned to send auditory information to motor centers that participate in prey capture and predator avoidance behaviors, such as the intermediate and deep layers of the superior colliculus, which controls head, eye, and pinnae movements for the orientation of objects in space [54]–[58]. Evidence also indicates that motor systems project to the IC, in which projections from the substantia nigra pars reticulata [59], [60] and globus pallidus [61] to the IC have been reported in rats. Additionally, lesions of the substantia nigra pars reticulata increase defensive responses (e.g., escape thresholds) induced by electrical or chemical stimulation of the IC [62]–[64]. In the present study, we compared outcomes when the stimuli (loud sounds) were immediately available to the IC for sensory processing (AEPs) and action outcomes when cortical or limbic processing of the signal was also required (EPM). We previously showed that conditioned fear, which recruits the mesocorticolimbic system, was significantly impaired by the DA receptor antagonist sulpiride [19], [34], [51]. In contrast, when the stimulus was eligible for IC processing as in the present study, the role of DA neurons associated with aversive situations that activated afferent sensory pathways via the IC appeared to be primary and possibly instrumental, and sulpiride increased the aversiveness of these stimuli. Thus, unconditioned and conditioned responses were differentially affected by the pharmacological manipulations. Indirect support for these results was previously provided by studies that demonstrated that systemic injections of the DA receptor antagonist sulpiride increased the switch-off response to light presented as an aversive US and enhanced fear-like behavior in the open arms of the EPM [17], [65]. Moreover, our recent study found that systemic administration of haloperidol strongly reduced ultrasonic vocalizations emitted during the testing session of a contextual fear conditioning paradigm [66], but increased the magnitude of AEPs in response to the unconditioned auditory aversive stimulation [16].

The startle reflex test measures a motor response whereas the AEPs recordings from electrodes directly implanted into the IC reflect the processing of sensory information. Depending on the aversive condition the startle reflex may be even reduced when the acoustic evoked potentials are enhanced [27]. Sensorimotor filtering is commonly activated in the midbrain tectum when acoustic or visual stimuli are presented to animals. As an explanation for the present findings, ascending projections from the IC would be first activated by aversive stimuli, and descending pathways would be sequentially recruited. This alternate circuit is associated with the processing of auditory information of an aversive nature, which triggers fear-like behavior [64], [67]. In fact, the IC is a key pathway for auditory information, and disturbances at this level may alter transmission to cortical centers. Abnormal cortical areas are known to exist in schizophrenia patients and may account for the abnormal processing of auditory information that results in auditory hallucinations and decreased responsiveness to sounds [68]. Interestingly, the lack of effect of intra-IC sulpiride administration on the startle reflex in response to noise alone is also consistent with the notion that the IC is not part of direct startle reflex circuitry [69], [70].

In summary, the present results show that D2-like receptors of the IC plays a role in the defensive behavior displayed by rats subjected to the elevated-plus maze, but not to the fear-potentiated startle test. Also, the reactivity of the neural substrates of fear in the inferior colliculus measured by AEPs to loud sounds is enhanced by the D2 receptor antagonist sulpiride locally injected into this structure. Dopamine appears to regulate unconditioned fear at the IC level, likely by reducing the sensorimotor gating of aversive events. Taking also into account that much evidence has shown that dopamine plays a role in the mediation of conditioned fear in the amygdala and nucleus accumbens the theory proposed by McNaughton and Corr [71] needs to be considered in future studies in this line of research. They consider that there is a rostrocaudal gradient in the brain, with unconditioned fear mapping at more caudal structures, such as the IC and dPAG, whereas conditioned fear is mapped at more rostral levels, such as the amygdala and prefrontal cortex. Thus, because the density of D_2_ receptors appears to largely predominate over other DA receptors in the IC [53], the potential use of the IC as a target to investigate unconditioned fear processes associated with DA mechanisms that are mediated by these receptors is unique. The present findings may stimulate further studies from this and other laboratories to provide further data on the differential role of DA in conditioned fear and the processing of unconditioned aversive information.

## Supporting Information

File S1 **Supporting figures.** **Figure S1, Table summary of the effects of** ***D_2_ drugs on the complementary categories of the elevated plus-maze.*** Effects of intra-IC injections of vehicle, quinpirole 1.0 µg/0.2 µL or 2.0 µg/0.2 µL and vehicle and 1.0, 2.0 or 4.0 µg/0.2 µL sulpiride on the complementary ethological categories of rats submitted to the elevated plus-maze. **Figure S2, Table summary of the effects of** ***D_1_ drugs on the complementary categories of the elevated plus-maze.*** Effects of intra-IC injections of vehicle, 1.0 or 2.0 µg/0.2 µL SKF-38393, and vehicle or 1.0, 2.0 or 4.0 µg/0.2 µL SCH-23390 on the complementary ethological categories of rats submitted to the elevated plus-maze. Figure S3, ***D_1_ drugs in the elevated plus-maze (EPM).*** Effects of intra-IC injections of vehicle, 1.0 and 2.0 µg/0.2 µL SKF-38393 or vehicle, 1.0, 2.0 or 4.0 µg/0.2 µL SCH-23390 on exploratory behavior of rats submitted to the elevated plus-maze. (A and D) Number of entries in the closed arms of the maze. (B and E) Number of entries in the open arms of the maze. (C and F). % of time spent into the open arms in relation to total. Figure S4, ***Fos-positive immunohistochemistry in response to aversive acoustic stimuli (AAS).*** Number of Fos-positive cells in midbrain (A) and telencephalic (B) structures in rats exposed to testing sessions with or without (Control) presentation of AASs. **Figure S5,** ***Fos-positive immunohistochemistry in midbrain structures in response to aversive acoustic stimuli (AAS).*** Photomicrographs of Fos-positive cells (dark dots) in the dmPAG, dlPAG, vlPAG, and IC in rats exposed to testing sessions with and without (Control) AAS presentation. **Figure S6,** ***Fos positive immunohistochemistry in telencephalic structures in response to aversive acoustic stimuli (AAS).*** Photomicrographs of Fos-positive cells (dark dots) in the Cg1, CPu, NAcC and NAcSh of rats exposed to testing sessions with (AAS) or without (Control) AAS presentation.
(PDF)
