# Supplementary material for: Dopamine D2-Like Receptors Modulate Unconditioned Fear: Role of the Inferior Colliculus
Source: PLoS One. 2014 Aug 18;9(8):e104228. doi: 10.1371/journal.pone.0104228 (PMC4136794; doi:10.1371/journal.pone.0104228)
Supplement: File S1 — Supporting figures. Figure S1, Table summary of the effects of D2 drugs on the complementary categories of the elevated plus-maze. Effects of intra-IC injections of vehicle, quinpirole 1.0 µg/0.2 µL or 2.0 µg/0.2 µL and vehicle and 1.0, 2.0 or 4.0 µg/0.2 µL sulpiride on the complementary ethological categories of rats submitted to the elevated plus-maze. Figure S2, Table summary of the effects of D1 drugs on the complementary categories of the elevated plus-maze. Effects of intra-IC injections of vehicle, 1.0 or 2.0 µg/0.2 µL SKF-38393, and vehicle or 1.0, 2.0 or 4.0 µg/0.2 µL SCH-23390 on the complementary ethological categories of rats submitted to the elevated plus-maze. Figure S3, D1 drugs in the elevated plus-maze (EPM). Effects of intra-IC injections of vehicle, 1.0 and 2.0 µg/0.2 µL SKF-38393 or vehicle, 1.0, 2.0 or 4.0 µg/0.2 µL SCH-23390 on exploratory behavior of rats submitted to the elevated plus-maze. (A and D) Number of entries in the closed arms of the maze. (B and E) Number of entries in the open arms of the maze. (C and F). % of time spent into the open arms in relation to total. Figure S4, Fos-positive immunohistochemistry in response to aversive acoustic stimuli (AAS). Number of Fos-positive cells in midbrain (A) and telencephalic (B) structures in rats exposed to testing sessions with or without (Control) presentation of AASs. Figure S5, Fos-positive immunohistochemistry in midbrain structures in response to aversive acoustic stimuli (AAS). Photomicrographs of Fos-positive cells (dark dots) in the dmPAG, dlPAG, vlPAG, and IC in rats exposed to testing sessions with and without (Control) AAS presentation. Figure S6, Fos positive immunohistochemistry in telencephalic structures in response to aversive acoustic stimuli (AAS). Photomicrographs of Fos-positive cells (dark dots) in the Cg1, CPu, NAcC and NAcSh of rats exposed to testing sessions with (AAS) or without (Control) AAS presentation. (PDF) [file pone.0104228.s001.pdf]

SUPPLEMENTARY MATERIAL (S1)

Figure S1. ***D_2 drugs in the complementary categories of the elevated plus-maze.*** Effects of intra-IC injections of vehicle (Control; n = 8), 0.5 µg/0.2 µL quinpirole (n = 7), 1.0 µg/0.2 µL quinpirole (n = 10), or 2.0 µg/0.2 µL quinpirole (n = 8), or vehicle (Control; n = 11), 1.0 µg/0.2 µL sulpiride (n = 10), 2.0 µg/0.2 µL sulpiride (n = 9), or 4.0 µg/0.2 µL sulpiride (n = 9), on the complementary ethological categories of rats submitted to the elevated plus-maze.

	D_2							
	Quinpirole				Sulpiride			
	control	qui0.5	qui1.0	qui2.0	control	sul1.0	sul2.0	sul4.0
**Grooming**	5.5 (1.4)	3.1 (0.9)	5.5 (1.8)	6.5 (1.1)	2.3 (0.8)	2.1 (0.7)	2.7 (0.5)	**6.2 (1.2)***
**Scanning**	49.8 (2.1)	47.6 (3.3)	42.0 (3.0)	43.1 (2.3)	53.3 (2.1)	50.3 (1.8)	49.4 (4.2)	51.4 (2.9)
**Head dipping**	12.8 (1.8)	12.3 (2.0)	7.0 (1.9)	8.1 (1.5)	11.5 (1.8)	13.7 (1.9)	7.7 (1.1)	9.0 (2.1)
**SAP**	2.8 (0.7)	2.4 (0.6)	3.2 (0.7)	2.5 (0.8)	2.7 (0.5)	3.4 (0.5)	3.0 (0.6)	4.7 (1.0)
**FBA**	2.1 (0.4)	1.3 (0.3)	0.9 (0.3)	1.3 (0.3)	1.9 (0.4)	1.1 (0.3)	1.0 (0.4)	0.7 (0.3)
**Rearing**	17.4 (1.6)	14.7 (1.8)	**11.5 (1.0)***	**10.8 (1.3)***	16.8 (1.3)	15.2 (2.1)	13.9 (2.0)	12.7 (1.4)
**EAE**	3.1 (1.1)	1.7 (0.5)	0.8 (0.4)	1.4 (0.5)	2.5 (0.7)	1.8 (0.6)	**0.6 (0.3)***	**0.8 (0.5)***
**Peeping out**	6.6 (1.7)	4.9 (0.7)	6.1 (1.3)	3.4 (0.6)	3.1 (0.4)	4.4 (0.7)	5.2 (0.8)	2.9 (0.6)

* $p < 0.05$, compared with control group (Newman–Keuls test). SAP, Stretched-attend posture; FBA, Flat-back approach; EAE, End-arm exploration.

Figure S2. ***D_1 drugs in the complementary categories of the elevated plus-maze.*** Effects of intra-IC injections of vehicle (Control; n = 10), 1.0 µg/0.2 µL SKF-38393 (n = 9), or 2.0 µg/0.2 µL SKF-38393 (n = 8), and vehicle (Control; n = 17), 1.0 µg/0.2 µL SCH-23390 (n = 10), 2.0 µg/0.2 µL SCH-23390 (n = 12), or 4.0 µg/0.2 µL SCH-23390 (n = 10), on the complementary ethological categories of rats submitted to the elevated plus-maze.

	D_1						
	SKF-38393			SCH-23390			
	**control**	**skf1.0**	**skf2.0**	**control**	**sch1.0**	**sch2.0**	**sch4.0**
**Grooming**	6.7 (0.9)	4.6 (0.8)	5.1 (1.2)	7.8 (1.1)	4.9 (1.7)	6.4 (1.5)	6.8 (1.6)
**Scanning**	51.5 (1.2)	51.3 (2.9)	54.0 (2.9)	54.0 (1.9)	53.0 (3.2)	50.6 (3.3)	55.7 (2.9)
**Head dipping**	11.4 (1.9)	11.6 (2.1)	15.2 (3.5)	11.2 (1.2)	13.7 (2.6)	7.5 (2.1)	13.3 (1.4)
**SAP**	4.4 (0.5)	4.3 (0.4)	4.4 (0.9)	3.4 (0.7)	4.0 (0.7)	3.3 (0.5)	3.8 (0.5)
**FBA**	1.0 (0.4)	0.6 (0.3)	0.4 (0.2)	0.9 (0.3)	0.2 (0.2)	0.2 (0.1)	1.2 (0.3)
**Rearing**	16.6 (1.8)	17.5 (2.3)	19.2 (1.1)	18.6 (1.5)	17.8 (2.5)	16.3 (1.5)	13.6 (1.8)
**EAE**	1.6 (0.5)	1.1 (0.6)	1.6 (0.9)	1.1 (0.3)	1.3 (0.5)	0.9 (0.5)	1.1 (0.3)
**Peeping out**	4.9 (0.8)	5.6 (0.8)	4.2 (0.5)	4.9 (0.6)	4.4 (0.6)	4.6 (0.5)	4.5 (0.6)

SAP: stretched attend postures. FBA: Flat-back approach. EAE: End-arm exploration

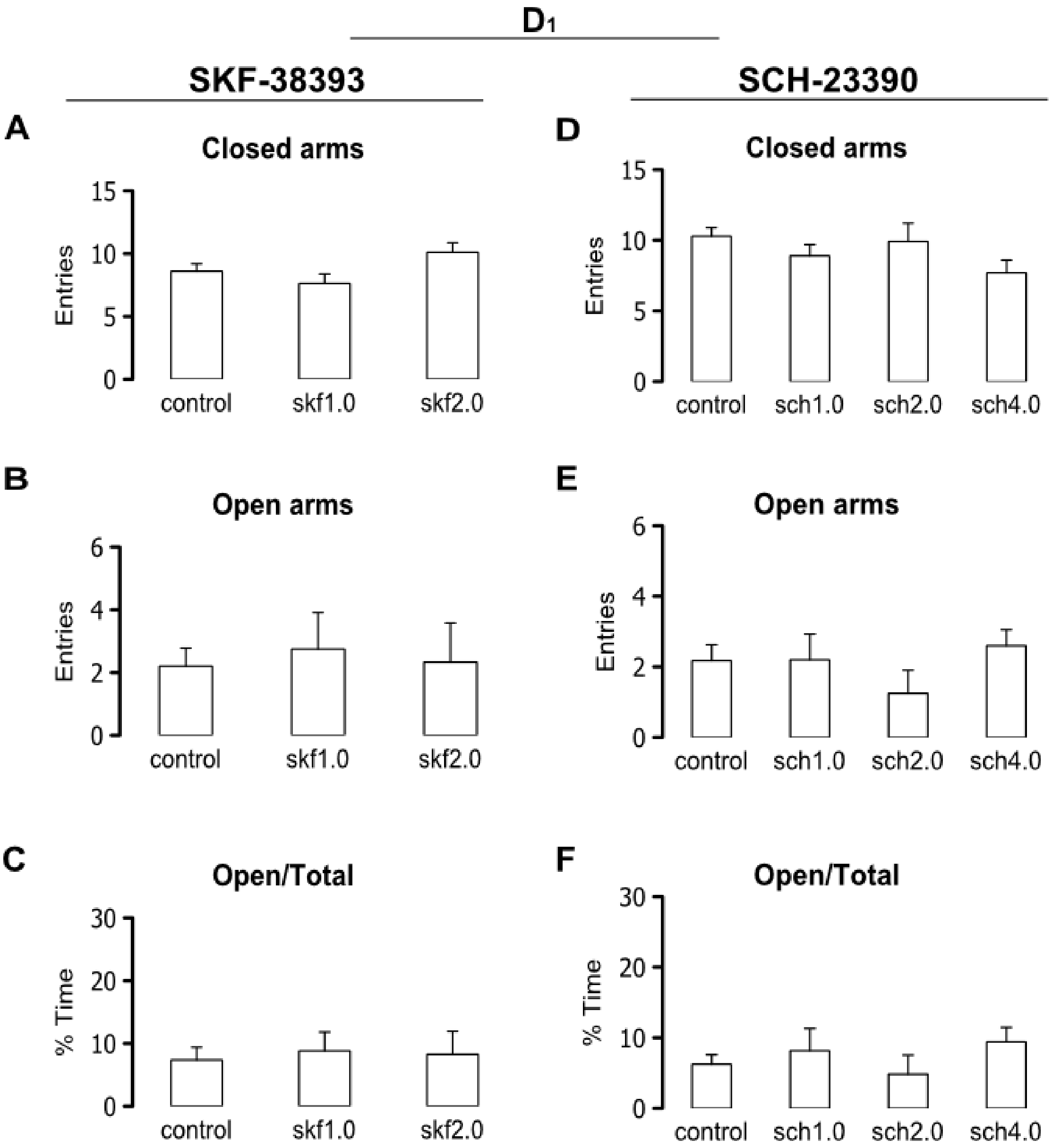

Figure S3. ***D_1 drugs in the elevated plus-maze (EPM).*** Effects of intra-IC injections of vehicle (Control; n = 10), 1.0 µg/0.2 µL SKF-38393 (n = 9), or 2.0 µg/0.2 µL SKF-38393 (n = 8), or vehicle (Control; n = 17), 1.0 µg/0.2 µL SCH-23390 (n = 10), 2.0 µg/0.2 µL SCH-23390 (n = 12), or 4.0 µg/0.2 µL SCH-23390 (n = 10), on exploratory behavior of rats submitted to the elevated plus-maze. (A and D) Number of entries in the closed arms of the maze. (B and E) Number of entries in the open arms of the maze. (C and F). % of time spent into the open arms in relation to total. The data are expressed as mean + SEM.

Immunohistochemical method

Fos protein immunohistochemistry. For this experiment, two groups of animals were used: exposed to AAS and not exposed to AAS (control). The AAS was presented as described in the AEP procedure above. Two hours after AAS or sham stimulation, the animals were deeply anesthetized with urethane (3 g/kg, i.p.) and transcardially perfused with 0.1 M phosphate-buffered saline (Na_2HPO_4 + $NaH_2PO_4 \cdot H_2O$ + NaCl + distilled H_2O) followed by 4% paraformaldehyde in 0.1 M PBS (pH 7.4). The brains were removed, immersed in paraformaldehyde for 2 h, and kept in 30% sucrose in 0.1 M PBS until soaked for cryoprotection. They were then quickly frozen in isopentane (−40°C) and sliced in a cryostat (−19°C). A series of 40 µm thick brain slices was obtained, with the following anterior/posterior coordinates as reference: bregma +2.28, +2.04, +1.20, -7.92, and -8.64 mm. The brain slices were collected in 0.1 M PBS and subsequently processed free-floating according to the avidin-biotin procedure using the Vectastain ABC Elite peroxidase rabbit IgG kit (ref. PK 6101; Vector Laboratories, Burlingame, CA, USA). The slices were first incubated with 1% H_2O_2 for 10 min, washed four times with 0.1 M PBS (5 min each), and then incubated overnight with the primary Fos polyclonal antibody (catalog no. SC-52; Santa Cruz Biotechnology, Santa Cruz, CA, USA) at a concentration of 1:2000 in PBS+ (0.1 M PBS enriched with 0.2% Triton-X and 0.1% bovine serum albumin [BSA]). The slices were washed again three times (5 min each) with 0.1 M PBS and incubated for 1 h with biotinylated goat antirabbit antibody (H + L; Vecstasin, Vector Laboratories, Burlingame, CA, USA) at a concentration of 1:400 in PBS+. After another series of three 5-min washes in 0.1 M PBS, they were incubated for 1 h with the avidin-biotin-peroxidase complex in 0.1 M PBS (A and B solution of the Vectastain ABC kit, Vector Laboratories, Burlingame, CA, USA) at a concentration of 1:250 in 0.1 PBS and washed three times in 0.1 M PBS. Fos immunoreactivity was revealed by the addition of the chromogen 3,3'-di-aminobenzidine (DAB; 0.02%) to which hydrogen peroxide (0.04%) was added just prior to use, followed by two washes with 0.1 M PBS.

Quantification of Fos-positive cells. Tissue sections were mounted on gelatin-coated slides and dehydrated for observation and cell counting under bright-field microscopy. Neuronal nuclei that expressed levels of DAB reaction product that were above tissue background were automatically counted as Fos-positive neurons by a computerized image analysis system (Image Pro Plus 4.0, Media Cybernetics, Bethesda, MD, USA) according to the method used previously in this laboratory [31-34].

Fos-positive immunohistochemistry results

S4 shows the number of Fos-positive cells in rats exposed to AASs (n = 7) and not exposed to AASs (Control, n = 7). Student's t-test revealed that exposure to AASs significantly increased the number of Fos-positive cells in the dorsomedial PAG ($t = 2.38$, $p < 0.05$), dorsolateral PAG ($t = 2.37$, $p < 0.05$), lateral PAG ($t = 2.22$, $p < 0.05$), ventrolateral PAG ($t = 2.26$, $p < 0.05$), and IC ($t = 2.59$, $p < 0.05$). The AAS did not produce changes in immunoreactivity in the cingulate cortex area 1 ($t = 0.08$, $p > 0.05$), caudate putamen ($t = 0.50$, $p > 0.05$), nucleus accumbens core ($t = 0.08$, $p > 0.05$), nucleus accumbens shell ($t = 1.49$, $p > 0.05$), or substantia nigra ($t = 0.62$, $p > 0.05$). Representative photomicrographs of Fos immunoreactivity are shown in S5 and S6.

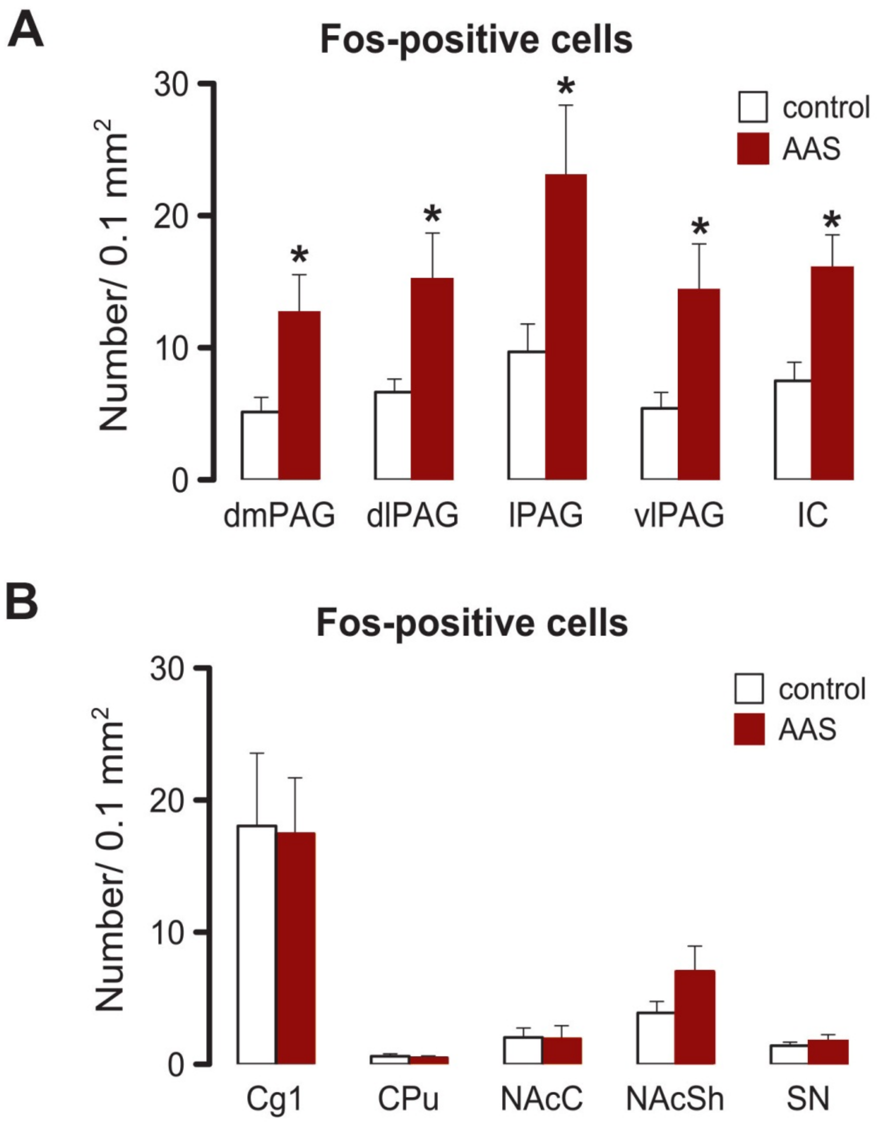

Figure S4. ***Fos-positive immunohistochemistry in response to aversive acoustic stimuli (AAS).*** Number of Fos-positive cells in midbrain (A) and telencephalic (B) structures in rats exposed to testing sessions with (AAS; n = 7) or without (Control; n = 7) presentation of AASs. The data are expressed as mean + SEM. *$p < 0.05$, compared with control group (Student's t-test). dmPAG, dorsomedial periaqueductal gray; dlPAG, dorsolateral periaqueductal gray; lPAG, lateral periaqueductal gray; vlPAG, ventrolateral periaqueductal gray; IC, inferior colliculus; Cg1, cingulate cortex area 1; CPu, caudate putamen (striatum); NAcC, nucleus accumbens core; NAcSh, nucleus accumbens shell; SN, substantia nigra.

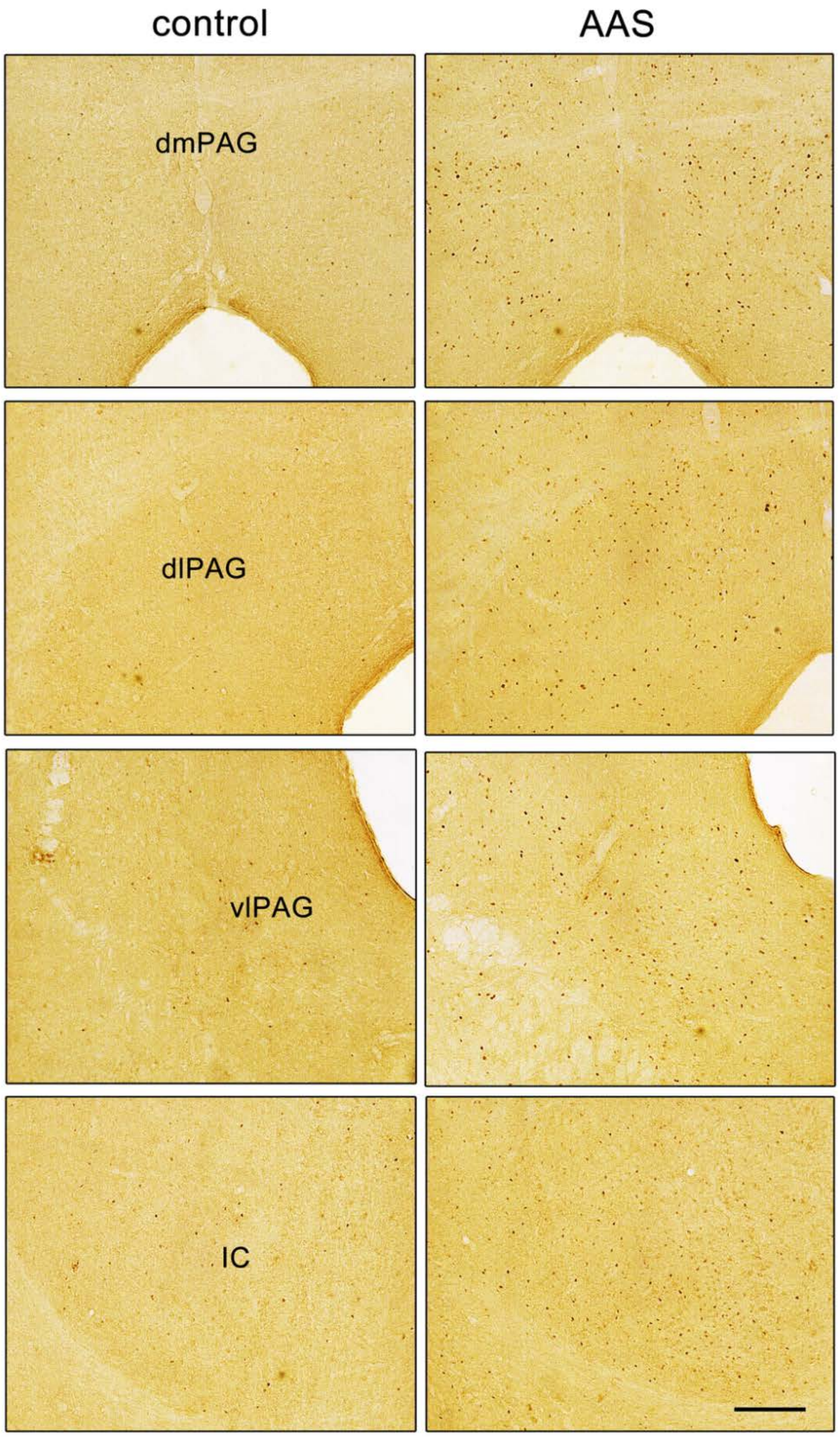

Figure S5. ***Fos-positive immunohistochemistry in midbrain structures in response to aversive acoustic stimuli (AAS).*** The figure shows photomicrographs of Fos-positive cells (dark dots) in the dmPAG, dlPAG, vlPAG, and IC in rats exposed to testing sessions with (AAS) and without (Control) AAS presentation. Scale bar = 200 µm. dmPAG, dorsomedial periaqueductal gray; dlPAG, dorsolateral periaqueductal gray; vlPAG, ventrolateral periaqueductal gray; IC, inferior colliculus.

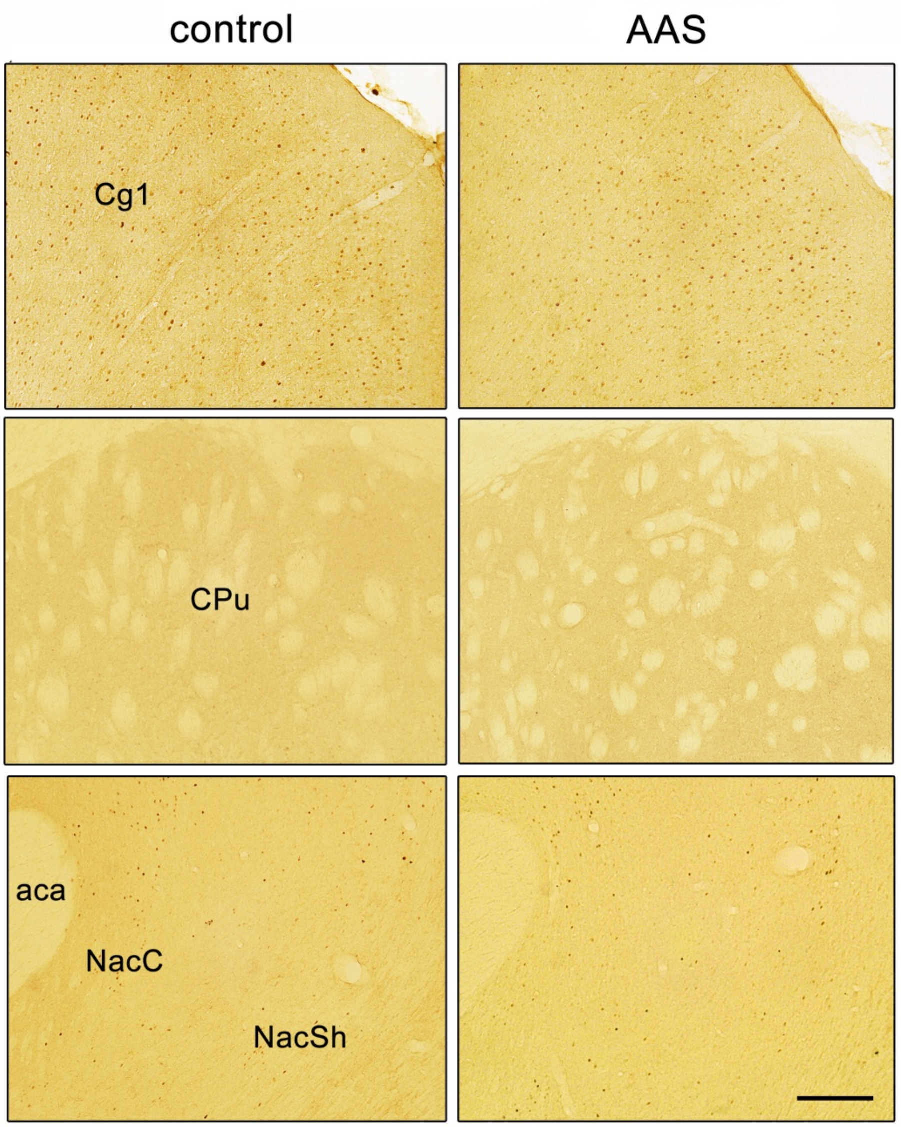

Figure S6. ***Fos positive immunohistochemistry in telencephalic structures in response to aversive acoustic stimuli (AAS).*** Photomicrographs of Fos-positive cells (dark dots) in the Cg1, CPu, NAcC and NAcSh of rats exposed to testing sessions with (AAS) or without (Control) AAS presentation. Scale bar = 200 μm. Cg1, cingulate cortex area 1; CPu, striatum; NAcC, nucleus accumbens core; NAcSh, nucleus accumbens shell.
